# Safety and immunogenicity of experimental stand-alone trivalent, inactivated Sabin-strain polio vaccine formulations in healthy infants: A randomized, observer-blind, controlled phase 1/2 trial

**DOI:** 10.1016/j.vaccine.2020.05.081

**Published:** 2020-07-14

**Authors:** Jakob P. Cramer, José Jimeno, Htay Htay Han, Stella Lin, Katharina Hartmann, Astrid Borkowski, Xavier Sáez-Llorens

**Affiliations:** a Takeda Pharmaceuticals International AG, Zurich, Switzerland; b Department of Infectious Diseases at Hospital del Niño Dr. José Renán Esquivel, Sistema Nacional de Investigación at SENACYT, Centro de Vacunación Internacional (Cevaxin), Panama City, Panama, USA; c Takeda Vaccines, Inc., Cambridge, USA

**Keywords:** Poliovirus, Inactivated vaccine, Sabin, Infnats, Toddlers

## Abstract

**Background:**

To increase the global supply of affordable IPV vaccine, preferably using Sabin viruses to comply with GAPIII requirements, Takeda has assessed three dosages of a stand-alone sIPV.

**Methods:**

In this phase I/II study two cohorts of 40 adults and 60 toddlers, respectively, were initially assessed for safety after receiving *high*-dosage sIPV compared with placebo (adults) or Salk IPV (toddlers). A cohort of 240 infants was then enrolled and randomized (1:1:1:1) to receive *low*-, *medium*- or *high*-dosage sIPV, or a reference Salk IPV in a three-dose primary schedule at 6, 10 and 14 weeks of age. Parents completed safety diaries for 4 weeks after each dose, and immunogenicity was measured as neutralization antibody titers at baseline and four weeks after vaccination.

**Results:**

All vaccinations were generally well-tolerated and sIPV had a comparable safety profile to the control arm in adults or the reference Salk IPV vaccine in toddlers and infants. Infants displayed dosage-dependent immune responses to sIPV when assayed using Sabin strains, which were equivalent to the reference IPV in the *high*-dosage sIPV group for serotypes 1 and 2, but not for Sabin and Salk serotype 3. Seroconversion rates (SCR) of the *low*- and *medium*-dosage groups were significantly lower than the Salk IPV group for both Sabin and Salk serotypes 1 and type 2 (p < 0.05), with no significant differences for Salk or Sabin serotypes 3. Responses to sIPV, particularly to Sabin types 1 and 2, were higher in initially seronegative infants, indicating possible interference by maternally-derived antibodies.

**Conclusions:**

A novel stand-alone Sabin-based IPV vaccine was well tolerated with an acceptable safety profile, but less immunogenic than reference Salk IPV at 6, 10 and 14 weeks of age for Salk serotypes 1 and 2, with apparent interference by maternal antibodies. Additional preclinical assessments will be made before any further clinical development.

## Highlights

• A novel IPV vaccine was developed from attenuated Sabin poliovirus strains.
• The vaccine was generally well tolerated in adults, toddlers and infants.
• The vaccine was immunogenic as a booster in previously polio-vaccinated toddlers.
• The vaccine was insufficiently immunogenic from 6 weeks of age in the EPI schedule.
• There was evidence of interference of the immune response by maternal antibodies.

## 1 Introduction

The world is close to eradicating the global scourge of wild polioviruses (WPV). One of the three poliovirus types, WPV type 2 was declared eradicated on September 20, 2015 [1], and the last case of WPV type 3 infection was identified in November 2012 and declared to be eradicated in October 2019 [2]. The only known WPV infections currently are due to type 1 virus with clinical cases occurring only in Afghanistan and Pakistan [3]. Most paralytic poliomyelitis cases around the world are no longer caused by WPV but are due to excreted viruses from live oral polio vaccines (OPV) which have reacquired neurovirulence, becoming circulating vaccine-derived polioviruses (cVDPV) [4]. In 2019 there were 329 cVDPV cases, mainly reported in Angola (114), the Central African Republic (19), the Democratic Republic of the Congo (84), Ethiopia (11), Ghana (12), Nigeria (18), Pakistan (22) and the Philippines (15) due to all three serotypes, compared with 173 WPV type 1 cases in Afghanistan (29) and Pakistan (144) [3].

With the eradication of WPV type 2 and the ongoing threat of cVDPV the WHO Strategic Advisory Group of Experts on Immunization working group (SAGE) recommended the removal of type 2 poliovirus from trivalent live poliovirus vaccines (tOPV) and a global switch to bivalent OPV vaccines (bOPV) [5]. To ensure some degree of ongoing immune coverage of infants against type 2 virus, the recommendation included the use of at least one dose of trivalent inactivated polio vaccine (IPV). The global switch to bOPV was successfully implemented in April 2016, the first step in the global withdrawal of live polioviruses vaccines following global eradication, to be followed by a post-eradication period when all immunizations will be based on IPV. More recently, the eradication of WPV 3 has been declared making the global withdrawal of all OPV and substitution by IPV more imminent.

However, there is a global shortage of IPV supply and increased demand will exacerbate this situation, creating an urgent need to expand manufacturing capacity and decrease costs of IPV in both the short- and long-term [6]. Further, the GAPIII containment recommendations by the SAGE [7], which accompanied the recommendation to withdraw type 2, also require stringent precautions be taken to eliminate the possibility of environmental contamination with the live polioviruses, limiting manufacture with Salk viruses to developed countries with established infrastructure. This has led to the suggestion that attenuated Sabin strains used in OPV vaccines may be inherently safer for use in manufacturing IPV [8], [9]. Several sIPV candidates are in clinical development, some previously shown to be safe and immunogenic in adults [10], [11] and in infants [12], [13], [14], [15], [16], leading to licensure and extensive use of sIPV in China and Japan [17], [18].

With this goal in mind, Takeda agreed a technology transfer from the BIKEN Foundation (formerly the Japan Poliomyelitis Research Institute, JPRI) for viral seeds to develop a Sabin IPV (sIPV) and investigate novel manufacturing technologies to enhance production capacity. The first use of the Takeda sIPV, in a combination with a diphtheria-tetanus-acellular pertussis (DTaP) vaccine (TAK-361s) in 3–67 month-old children, has been reported [16]. This paper describes a Phase 1/2 dosage-ranging assessment of stand-alone formulations of sIPV (TAK-195) using the 6–10-14 week schedule currently used in many low- and middle-income countries.

## 2 Methods

### 2.1 Design and ethical approval

This Phase 1/2, randomized, observer-blind, safety and tolerability dose ranging trial was performed in three sequential stages at the Hospital del Niño Doctor José Renán Esquivel, Panama City, Panama from June 7, 2017 until its premature termination on October 18, 2018. The purpose of this study was to select the optimal antigen content of a stand-alone trivalent sIPV (TAK-195) consisting of the three Sabin poliovirus strains (types 1, 2, and 3) to be taken forward into further clinical development. The study protocol was approved by the hospital Bioethical Committee, registered on ClinicalTrials.gov (NCT03092791) and conducted in accordance with ICH-GCP and Declaration of Helsinki guidelines and applicable local regulatory requirements.

*Low*-, *medium*- and *high*-dosage sIPV candidate formulations were administered in a three-dose primary infant immunization series at 6, 10 and 14 weeks of age. Selection of the vaccine composition was to be based on a comparison of the safety and tolerability profile after each dose, and the immune responses to both Sabin and Salk strain poliovirus types 1, 2, and 3 after the third dose of this infant series. Vaccination of infants was only performed following approval by an independent Data Monitoring Committee (DMC) who considered the data from two successive single-dose safety lead-in stages in which the high-dosage formulation was administered to cohorts of adults and then toddlers, respectively.

The protocol originally envisaged assessment of immunogenicity in infants at Day 365, one year after completion of the primary series, and then the administration of a booster vaccination with the respective sIPV or reference IPV with assessment neutralizing antibodies 28 days later. Following the post-primary assessment of assessment of immunogenicity the study was terminated before these scheduled interventions.

### 2.2 Participants

For the first safety assessment forty adults (18–49 years of age) with a history of complete primary polio immunization were recruited and randomly assigned 1:1 to receive one injection of either the *high*-dosage sIPV or placebo. Following an assessment of 7-day safety data from these participants, the DMC gave approval for enrolment of a cohort of sixty toddlers (12–15 months of age) who were randomly assigned 1:1 to receive one vaccination with either the *high*-dose sIPV or a reference Salk IPV. Other routine vaccinations for the toddler age group were only allowed 4 weeks before or after these polio immunizations. The DMC evaluated 7-day safety data from this toddler cohort before they approved enrolment of the infant cohort of 240 children (6–8 weeks of age). Eligible infants were healthy, with no chronic medical conditions, and no prior history of polio vaccination.

### 2.3 Vaccines

The investigational vaccine formulations were manufactured using a novel proprietary bioreactor system by Takeda (Hikari, Japan). Final lots were prepared in single-dose vials and consisted of trivalent formulations of formaldehyde-inactivated Sabin poliovirus types 1, 2 and 3 with 0.3 mg aluminium as Al(OH)_3_ in 0.5 mL of 10 mM phosphate buffer containing 2.5 µL 2-phenoxyethanol. The three formulations were classified as *low-dosage*, containing 0.75, 25 and 25 D-antigen units (DU) of Sabin types 1, 2 and 3, respectively, *medium-dosage* (1.5, 50 and 50 DU) and *high-dosage* (3, 100 and 100 DU). Administration was by intramuscular injection, preferably in the right limb, with infant groups receiving a pentavalent (DTP-HBV-Hib) vaccine concomitantly in the opposite limb. The control groups of infants and toddlers received a commercial reference Salk IPV (Bilthoven Biologicals, Bilthoven, the Netherlands). Placebo for use in adults was 0.9% saline for injection. All administrations were performed by study personnel blinded to the identity of each injection.

### 2.4 Safety and reactogenicity

All participants were monitored for 30 min after each vaccination for any immediate reaction or temperature elevation. The co-primary safety objective was the safety and tolerability of each of the three immunizations with the sIPV formulations in the infant cohort. For this assessment (and in the toddler cohort) parents/guardians completed diary cards which solicited local reactions and systemic adverse events (AEs) as well as a daily temperature reading for 7 days after each of the three vaccinations, and any unsolicited AEs occurring before the next clinic visit. Any serious adverse event (SAE), defined as resulting in death or life threatening, or necessitating hospitalization, was to be reported immediately to the principal investigator and study sponsor during the entire study period.

### 2.5 Immunogenicity

The co-primary immunogenicity objective in the infant cohort was the WHO recommended parameter for assessment of new IPVs - the seroconversion rate for each of the three poliovirus types 28 days (day 85) after completion of the primary immunization series [19]. For this assessment blood was drawn on Days 1, 57 (for an interim assessment after two doses) and 85. Sera were stored at −20℃ for shipping to the Centers for Disease Control and Prevention, Atlanta, Georgia, USA, for measurement of poliovirus-specific neutralization activity using a standardized assay [20]. Neutralization titers for each of the poliovirus types were measured separately for Sabin and Salk strains in the assay.

No blood draws or immunogenicity assessments were performed in the adult cohort. Two blood samples were drawn from the toddler cohort, before vaccination on Day 1 and on Day 29, respectively, for an exploratory analysis to ensure these children displayed immune responses to these booster doses. Any toddler who had no antibodies against any serotype or did not display any titer increase after receiving sIPV vaccination was to be offered a further vaccination with the Salk IPV. Similarly, in the event that any infant did not achieve seroprotective levels against any poliovirus serotype parents would also be offered a catch-up vaccination for their child with the reference vaccine.

### 2.6 Statistics

The study was not powered for statistical comparisons, all comparisons being intended to be descriptive. In infants and toddler groups geometric mean titers (GMT) of neutralizing antibodies were calculated for all three serotypes for each group at each time point. Seropositivity/seroprotection rates (SPR) were defined as the percentages of infants or toddlers in each group with antibody titers ≥ 8 at the respective timepoint. In infants seroconversion rates were defined as group percentages in initially seronegative infants (titer < 8 at Day 1) having a titer ≥ 8 at Day 85, or initially seropositive infants (titer ≥ 8 at Day 1 presumed to be due to maternal antibodies) displaying a ≥ 4-fold rise in antibody titers over the expected level of maternal antibodies at Day 85, calculated using a decline in maternal antibody titers with an assumed half-life of 28 days. Post hoc calculations of the differences between seroconversion rates in the sIPV and reference Salk IPV groups were performed by the Newcombe method [21], with p values using Fishers Exact Test.

## 3 Results

### 3.1 Demographics

Demographics of the enrolled adult, toddler and infant cohorts are shown in Table 1. Apart from some variations in the gender and ethnicity ratios in the adult groups, there were similar distributions in terms of age, gender and race across groups of adults, toddlers and infants.

**Table 1:** Demographics of the Adult, Toddler and Infant Per Protocol study populations.

		Adults		Toddlers		Infants			
		High dosage sIPV	Placebo	High dosage sIPV	Reference Salk IPV	Low dosage sIPV	Medium dosage sIPV	High dosage sIPV	Reference Salk IPV
**Enrolled**		N = 20	N = 20	N = 30	N = 30	N = 60	N = 60	N = 60	N = 60
**Per Protocol exclusions**	Enrolled but not vaccinated	–	–	–	–	1	–	–	–
	Had an exclusion criterion	–	–	3	1	–	–	1	–
	Issue with study medication	–	–	–	–	3	12	4	2
	Outside of visit window	–	–	–	–	–	–	1	2
	Death	–	–	–	–	–	1	–	1
**Per protocol population**		N = 20	N = 20	N = 27	N = 29	N = 56	N = 47	N = 54	N = 55
**Mean Age ± SD** *		34.9 ± 8.56	35.1 ± 8.23	14.0 ± 0.98	14.0 ± 1.02	6.6 ± 0.68	6.5 ± 0.66	6.6 ± 0.69	6.5 ± 0.60
**Male:female**		4:16	7:13	15:12	18:11	26:30	19:28	29:25	35:20
**Race** n (%)	American Indian/Alaskan Native	18 (90.0)	13 (65.0)	26 (96.3)	28 (96.6)	54 (96.4)	44 (93.6)	49 (90.7)	53 (96.4)
	Black / African American	1 (5.0)	6 (30.0)	1 (3.7)	1 (3.4)	1 (1.8)	2 (4.3)	2 (3.7)	2 (3.6)
	White	1 (5.0)	1 (5.0)	0	0	1 (1.8)	1 (2.1)	2 (3.7)	0
	Other	0	0	0	0	0	0	1 (1.9)	0
**Baseline seropositivity** n (%)	Sabin type 1	–	–	24 (88.9)	25 (86.2)	29 (51.8)	24 (51.1)	19 (35.2)	23 (41.8)
	Sabin type 2	–	–	25 (92.6)	27 (93.1)	39 (69.6)	34 (72.3)	35 (64.8)	36 (65.5)
	Sabin type 3	–	–	25 (92.6)	26 (89.7)	13 (23.2)	16 (34.0)	16 (29.6)	19 (34.5)
	Salk type 1	–	–	26 (96.3)	28 (96.6)	19 (33.9)	17 (36.2)	17 (31.5)	18 (32.7)
	Salk type 2	–	–	26 (96.3)	26 (89.7)	23 (41.1)	21 (44.7)	23 (42.6)	23 (41.8)
	Salk type 3	–	–	25 (92.6)	26 (89.7)	12 (21.4)	6 (12.8)	13 (24.1)	12 (21.8)

* Adults = years; Toddlers = months; Infants = weeks.

### 3.2 Safety assessments – Adults and toddlers

The two groups of twenty adults received *high-*dosage sIPV or placebo. The DMC considered that these vaccinations in adults displayed acceptable tolerability with no safety concerns (Table 2), allowing enrolment and vaccination of the toddler group.

**Table 2:** Incidence rates of solicited local reactions and systemic adverse events (AE) within 7 days of vaccination in adult and toddler Safety Sets.

		**Incidence, n subjects (%)**			
		**Adults**		**Toddlers**	
		High-dosage sIPV	Placebo	High-dosage sIPV	Reference Salk IPV
**Subjects with completed diary cards**		n = 20	n = 20	n = 30	n = 30
**Local reactions**					
Pain *	**Any**	9 (45)	4 (20)	10 (33.3)	4 (13.3)
	*Mild*	*7 (35)*	*4 (20)*	*8 (26.7)*	*3 (10.0)*
	*Moderate*	*2 (10)*	*0*	*1 (3.3)*	*1 (3.3)*
	*Severe*	*0)*	*0*	*1 (3.3)*	*0*
Erythema	Any	1 (5)	0	0	0
Induration	Any	0	1 (5)	0	0
Swelling	Any	0	1 (5)	0	0
**Systemic adverse events**					
Fever	≥ 38.0 °C	0	1 (5.0)	2 (6.9)	2 (6.9)
	≥ 40.0 °C	0	0	0	0
Headache	Any	3 (15.0)	3 (15.0)	na	na
Asthenia	Any	0	0	na	na
Malaise	Any	1 (5.0)	1 (5.0)	na	na
Arthralgia	Any	2 (10.0)	1 (5.0)	na	na
Myalgia	Any	4 (20.0)	3 (15.0)	na	na
Drowsiness	Any	na	na	6 (20.0)	6 (20.0)
Irritability	Any	na	na	4 (13.3)	7 (23.3)
Loss of appetite	Any	na	na	5 (16.7)	3 (10.0)
**SAEs throughout study**					
	**Any**	0	0	0	0

na: not applicable as not assessed in this age group.

Similarly, vaccinations in both groups of toddlers given either *high-*dosage sIPV or the Salk IPV were generally well tolerated, with no serious or severe AEs and no clinically relevant differences between groups (Table 2), so the DMC authorized enrolment of the infant cohort.

### 3.3 Safety assessments - infants

Of the 240 infants enrolled and randomized to the three groups, 239 (99.6%) received at least one dose of sIPV or Salk IPV and were included in the Safety Set (FAS) – one enrollee in the low-dosage group did not receive vaccine. Only solicited local reactions within 7 days of vaccination could be ascribed directly to the investigational sIPV formulations or reference Salk IPV, as it was not possible to attribute solicited systemic adverse events specifically to these vaccines rather than the concomitantly administered DTP-HBV-Hib vaccine. Across all doses, incidences of solicited local reactions to any IPV were consistent across groups; most were injection site pain, about 28% of which were described as severe. Other local reactions were mild to moderate (Table 3). The most frequent systemic AEs within 7 days of vaccination were drowsiness and irritability, the majority of which were mild to moderate, and occurred at similar rates in all groups, including the Salk vaccine recipients. Fever was reported after 18% of all Salk vaccine doses and 12% of all sIPV doses, but there was only one case of fever ≥ 40.0 °C, which occurred in the Salk vaccine group. Mild or moderate unsolicited adverse events occurred at similar rates 28 days after each dose in all groups and none was considered to be related to the vaccination (Table 4), with no effects of dose, formulation dosage, or sIPV or Salk IPV.

**Table 3:** Incidence rates of solicited local reactions and systemic adverse events (AE) within 7 days of any vaccination and SAEs throughput the study in infants from the Safety Set.

		**Incidence, n subjects (%)**			
		Dosage of sIPV			Reference
		Low	Medium	High	Salk IPV
**Subjects with completed diary cards**		n = 57	n = 52	n = 56	n = 57
**Local reactions**					
Pain *	**Any**	52 (91.2)	41 (78.8)	50 (89.3)	53 (93.0)
	*Mild*	*15 (26.3)*	*15 (28.8)*	*24 (42.9)*	*16 (28.1)*
	*Moderate*	*21 (36.8)*	*13 (25.0)*	*15 (26.8)*	*23 (40.4)*
	*Severe*	*16 (28.1)*	*13 (25.0)*	*11 (19.6)*	*14 (24.6)*
Erythema	**Any**	3 (5.3)	3 (5.8)	1 (1.8)	2 (3.5)
Induration	**Any**	1 (1.8)	2 (3.8)	0	2 (3.5)
Swelling	**Any**	2 (3.5)	4 (7.7)	0	1 (1.8)
**Systemic adverse events**					
Fever	≥ 38.0 °C	16 (28.1)	13 (25.0)	20 (35.7)	22 (38.6)
	≥ 40.0 °C	0	0	0	1 (1.8)
Drowsiness ^#^	**Any**	36 (63.2)	32 (61.5)	32 (57.1)	36 (63.2)
	*Mild*	*22 (38.6)*	*23 (44.2)*	*15 (26.8)*	*20 (35.1)*
	*Moderate*	*13 (22.8)*	*8 (15.4)*	*16 (28.6)*	*15 (26.3)*
	*Severe*	*1 (1.8)*	*1 (1.9)*	*1 (1.8)*	*1 (1.8)*
Irritability ^#^	**Any**	37 (64.9)	32 (61.5)	37 (66.1)	41 (71.9)
	*Mild*	*15 (26.3)*	*12 (23.1)*	*18 (32.1)*	*18 (31.6)*
	*Moderate*	*12 (21.1)*	*14 (26.9)*	*10 (17.9)*	*16 (28.1)*
	*Severe*	*10 (17.5)*	*6 (11.5)*	*9 (16.1)*	*7 (12.3)*
Loss of appetite ^#^	**Any**	22 (38.6)	16 (30.8)	18 (32.1)	16 (28.1)
	*Mild*	*19 (33.3)*	*14 (26.9)*	*11 (19.6)*	*7 (12.3)*
	*Moderate*	*3 (5.3)*	*2 (3.8)*	*6 (10.7)*	*8 (14.0)*
	*Severe*	*0*	*0*	*1 (1.8)*	*1 (1.8)*
**SAEs throughout study**					
	**Any**	8 (13.6)	5 (8.3)	5 (8.3)	8 (13.3)

* Pain was defined as mild (minor reaction to touch), moderate (cries/protests on touch) or severe (cries when limb moved/spontaneously painful). All cases of erythema, induration and swelling were mild or moderate.

# Definitions of mild, moderate and severe systemic AEs could be added here.

**Table 4:** Incidence rates of unsolicited adverse events (AE) within 28 days of any vaccination and serious adverse events (SAE) throughout the study duration in the infant Safety Set.

		**Incidence, n subjects (%)**			
		Dosage of sIPV			Reference
		Low	Medium	High	Salk IPV
**Subjects in Safety Set**		n = 59	n = 60	n = 60	n = 60
**After dose 1**	**Any Unsolicited AE**	20 (33.9)	11 (18.3)	12 (20.0)	15 (25.0)
	*Mild*	*18 (30.5)*	*8 (13.3)*	*11 (18.3)*	*11 (18.3)*
	*Moderate*	*2 (3.4)*	*3 (5.0)*	*1 (1.7)*	*4 (6.7)*
	*Severe*	*0*	*0*	*0*	*0*
	**SAE**	3 (5.1)	1 (1.7)	0	3 (5.0)
	Bronchiolitis/Bronchitis	2	–	–	2
	Pneumonia	–	1	–	–
	Aseptic meningitis	–	–	–	1
	Urinary tract infection	1	–	–	–
**After dose 2**	**Any Unsolicited AE**	12 (20.3)	17 (28.3)	14 (23.3)	15 (25.0)
	*Mild*	*12 (20.3)*	*15 (25.0)*	*11 (18.3*	*15 (25.0)*
	*Moderate*	*0*	*2 (3.3)*	*3 (5.0)*	*0*
	*Severe*	*0*	*0*	*0*	*0*
	**SAE**	0	0	1 (1.7)	0
	Pneumonia	–	–	1	–
**After dose 3**	**Any Unsolicited AE**	11 (18.6)	14 (23.3)	15 (25.0)	10 (16.7)
	*Mild*	*11 (18.6)*	*13 (21.7)*	*14 (23.3)*	*8 (13.3)*
	*Moderate*	*0*	*0*	*1 (1.7)*	*2 (3.3)*
	*Severe*	*0*	*1 (1.7)*	*0*	*0*
	**SAE**	5 (8.5)	4 (6.7)	4 (6.7)	5 (8.3)
	Death	–	1	–	1
	Cyanosis	–	–	1	–
	Milk allergy	1	–	–	–
	Pneumonia	1	3	2	2
	Gastroenteritis	2	–	–	–
	Bronchiolitis/Bronchitis	–	–	1	–
	Pharyngotonsillitis	–	–	–	1
	Tracheitis	1	–	–	–
	Seizure	–	–	–	1

There were 26 SAEs reported in infants which were all typical conditions occurring an infant population—mainly bronchiolitis and pneumonia—and none was considered to be related to vaccination (Table 4**)**. There were two deaths; one infant from the *medium*-dosage sIPV group suffered Sudden Infant Death syndrome two days after their third vaccinations, and a second infant in the Salk IPV group died of unknown cause 35 weeks after the last of the three primary vaccinations. Neither case was considered to be associated with the study vaccines or procedures.

### 3.4 Immunogenicity

**Fig. 1:**
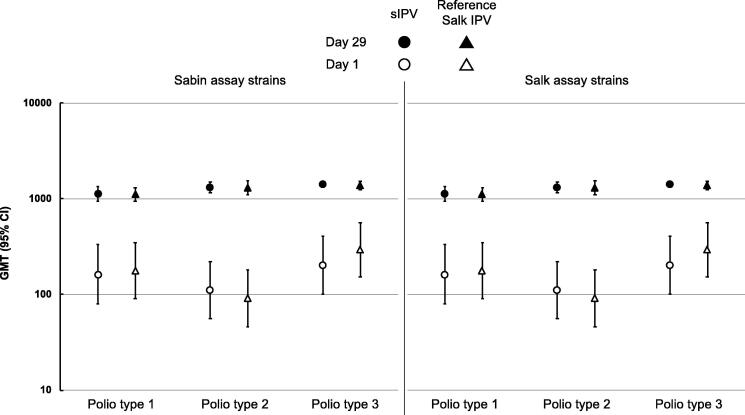
Geometric mean neutralizing antibody titers (with 95% CI) against the three Sabin (left panel) and three Salk (right panel) poliovirus strains at Day 1 before vaccination, and at Day 29 after one booster vaccination with high dose sIPV or reference Salk IPV in the two toddler groups (n = 20).

The two toddler groups had similar pre-vaccination serostatus, with 86.2–92.6% and 89.7–96.6% being seropositive for Sabin and Salk serotypes, respectively (Table 1). All toddlers (100%) were seropositive for all Sabin and Salk serotypes after vaccination with either sIPV or Salk IPV, with similar GMTs across groups for all Sabin and Salk serotypes (Fig. 1).

After excluding a further 25 infants from the Per Protocol population due to major protocol violations there were 212 infants (88.3%) eligible for immunogenicity assessment (Table 1). The violations mainly consisted of receipt of medications prohibited in the study protocol affecting 23 children (3, 13, and 4 in *low*-, *medium*- and *high*-dosage IPV groups, and 3 in the reference IPV group) and three children who were presented outside of the study visit window (1 in the *high*-dosage sIPV group and 2 in the reference IPV group) including one who received proscribed medication. In the infant cohort baseline seropositivity rates were 30.3–68.3% and 20.4–42.7% against Sabin and Salk serotypes, respectively, presumably due to placentally-transferred maternal antibodies in these unvaccinated children.

#### 3.4.1 Seroconversion rates

In the evaluated infant cohort (N = 212) responses assessed as the primary endpoint, seroconversion rates (SCR) after three vaccinations, were markedly different when measured using either Sabin or Salk poliovirus strains in the neutralization assay (Table 5). There were also clear differences in the profiles of the responses to poliovirus types 1 and 2 compared with those to type 3. There were dosage-dependent SCR responses induced by sIPV against the Sabin type 1 and 2 assay strains, while SCRs against the Sabin type 3 were similar in all four groups. For Sabin type 1 and 2 assay strains the SCRs were significantly lower in *low-* and *medium*-dosage sIPV groups compared with the Salk IPV vaccine group; the *low*-dosage sIPV group also had significantly lower SCRs than the *high-dosage* sIPV group for both types.

**Table 5:** Seroconversion rates in infants at Day 85 after three doses of *low*-, *medium*- or *high*-dose sIPV and reference Salk IPV for the three poliovirus types using either Sabin or Salk viruses in the neutralizing assays (Per Protocol population).

		**Seroconversion rate as n subjects per group (%)**			
		**Dosage of sIPV**			**ReferenceSalk IPV**
		Low	Medium	High	
**Assay strain**	N =	54	45	50	53
**Sabin** n seroconverted (**%**)	Type 1	34(**63.0**) ^b,x^	35(**77.8**) ^a^	43(**86.0**)	49(**92.5**)
	Type 2	37(**68.5**) ^b,y^	35(**77.**8) ^a^	45(**90.0**)	50(**94.3**)
	Type 3	52(**96.3**)	45(**100**)	48(**96.0**)	51(**96.**2)
**Salk** n seroconverted (**%**)	Type 1	20(**37.0**) ^b,x^	24(**53.3**) ^a^	34(**68.0**) ^b^	51(**96.2**)
	Type 2	19(**35.2**) ^x,z^	25(**55.6**) ^b^	35(**70.0**) ^b^	51(**96.2**)
	Type 3	49(**90.**7)	42(**93.3**)	47(**94.0**)	50(**94.3**)

^a^p < 0.05 and ^b^ p < 0.001 vs. reference Salk IPV by Fisher’s Exact test.

^x^ p < 0.05, ^y^ p < 0.01 and ^z^ p < 0.001 vs. high-dose sIPV by Fisher’s Exact test.

When responses were measured using the Salk strains in the assay the sIPV groups again demonstrated a dosage-dependent increase in SCR, Salk type 1 and 2 SCRs being significantly lower with *low-*dosage sIPV than with high-dosage sIPV, but all dosages of sIPV achieved significantly lower SCRs for Salk types 1 and 2 than the reference Salk IPV. With *high*-dosage sIPV the SCRs against Salk type 1 and 2 were 68.0% and 70.0%, respectively, compared with 96.2% against both types induced by Salk IPV. As observed when using the Sabin type 3 strain in the assay, SCRs when using the Salk type 3 strain were similar with all three sIPV dosages and the reference Salk IPV.

#### 3.4.2 Geometric mean titers (GMTs)

**Fig. 2:**
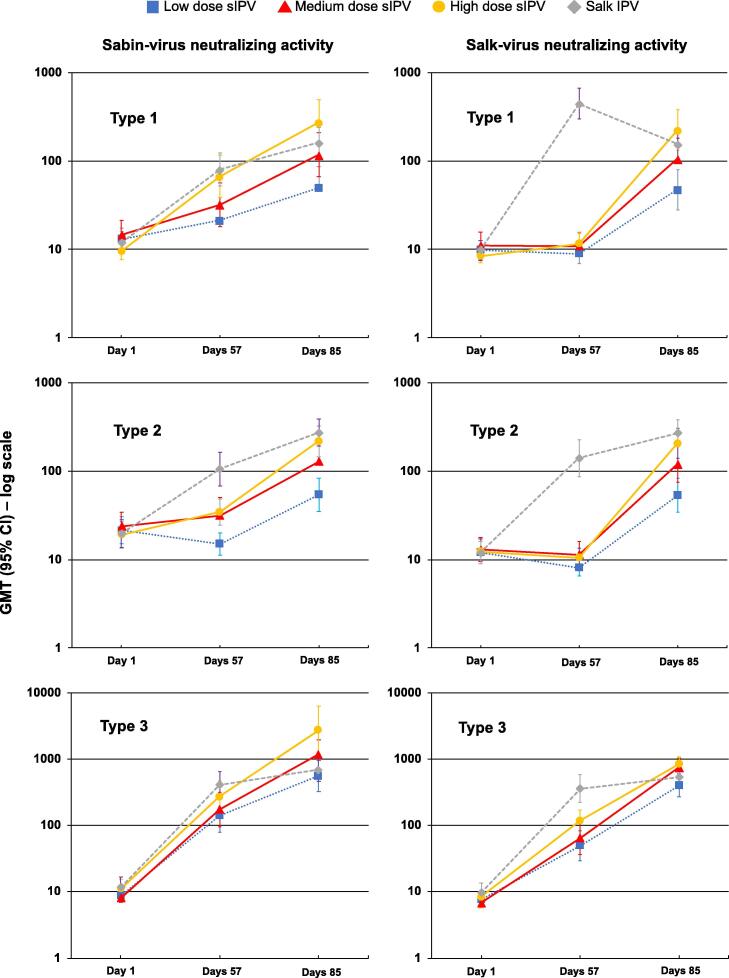
Geometric mean neutralizing antibody titers (with 95% confidence bars) against the three Sabin (left panels) and Salk (right panels) poliovirus serotypes at Day 1 before vaccination, and at Days 57 and 85 after two and three primary vaccinations in the four infant groups,

The dosage-response trend and lower responses to sIPV than Salk IPV for *low*-, *medium*- and *high*-dosages of sIPV for both Sabin and Salk types 1 and 2 assay strains were also clear when the responses were expressed as GMTs after 3 doses (Day 85), and particularly evident at the interim time point of day 56 four weeks after the second doses had been administered (Fig. 2). The final type 1 and 2 GMTs after the third *high*-dosage of sIPV were similar to those induced by three doses of the reference Salk IPV measured using either Sabin or Salk viruses in the assay. In the Salk IPV group there was a trend for type 1 and 2 GMTs to display the main increment of their increase after two doses, but in the three sIPV groups more important increments of the increases in GMTs occurred after the third dose.

GMT profiles for antibodies against type 3, using either Sabin or Salk viruses in the assay, were similar in all groups at each time-point with some indication of dosage-dependence.

#### 3.4.3 Seroprotection rates

**Fig. 3:**
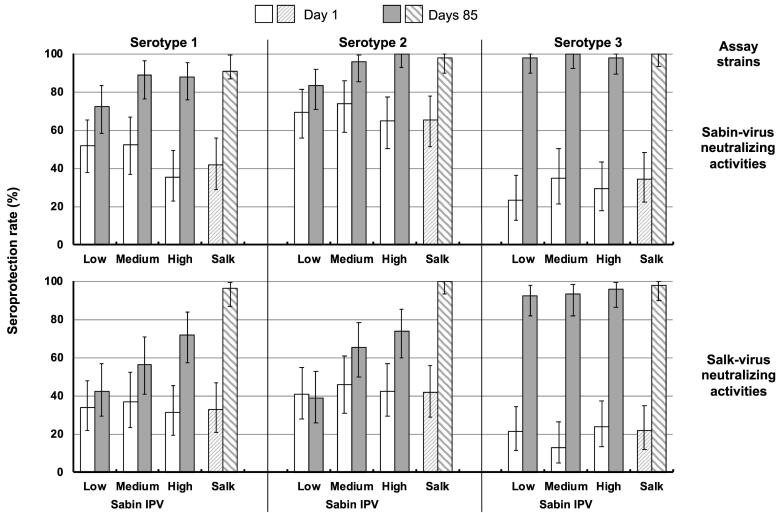
Group seroprotection rates (% with titers ≥ 8) using the three poliovirus Sabin serotypes in the assay (upper panels) and the three Salk serotypes in the assay (lower panels) at Day 1 before vaccination and Day 85 after completion of the three primary vaccinations in the four infant groups according to vaccine dosage. Values shown as means with 95% confidence bars.

Before vaccination 44.8%, 67.9% and 30.2% of the infant cohort were seropositive against Sabin types 1, 2 and 3, respectively, and 33.5%, 42.5% and 20.3% were seropositive against Salk types 1, 2 and 3 (Fig. 3). After the third vaccination seroprotection rates against Sabin strain types 1 were similar for *medium*- and *high*-dosage sIPV and Salk IPV groups (89.1% 88.0% and 96.2%, respectively), and slightly lower for the *low*-dosage sIPV group (72.2%). Similarly, seroprotection rates measured against Sabin type 2 were 83.3%, 95.7%, 100% and 98.1% for *low*-, *medium*- and *high*-dosage sIPV and Salk IPV groups, respectively, and for Sabin type 3 rates were 98.0–100% across groups (Fig. 3).

Seroprotection rates measured against Salk strains showed a clear dosage-dependence in the sIPV groups for types 1 and 2, but with levels below those achieved in the Salk IPV group (Fig. 3). The high responses induced by all three dosages of sIPV against type 3 were also evident when measured with Salk virus, with rates of 92.6%, 93.5%, 96.0% and 98.1% for *low*-, *medium*- and *high*-dosage sIPV and Salk IPV groups, respectively.

#### 3.4.4 Influence of prior serostatus

**Fig. 4:**
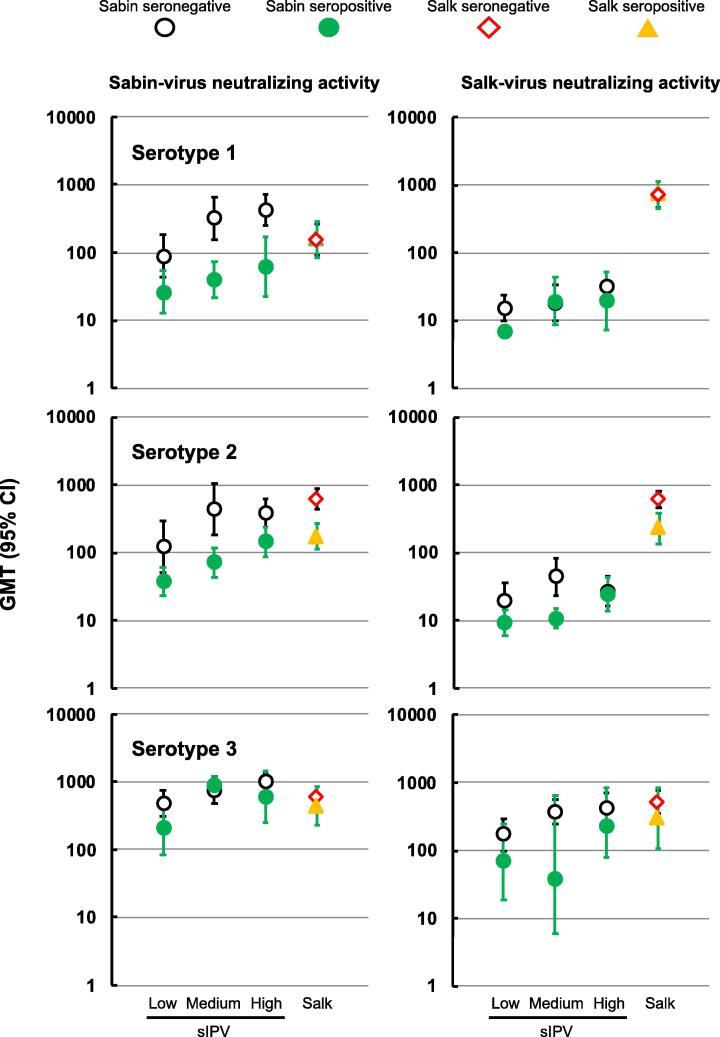
Geometric mean neutralizing antibody titers (with 95% confidence bars) at Day 85 against the three poliovirus serotypes as Sabin (upper panel) and Salk strains (lower panel) after completion of the three primary vaccinations according to pre-vaccination serostatus in the four infant groups.

When Day 85 GMTs were assessed according to the prevaccination serostatus of the participants using Sabin viruses in the assay the responses induced by sIPV against types 1 and 2 were markedly lower in initially seropositive infants, suggesting interference by maternal antibodies (Fig. 4). Responses against Sabin type 3 virus were unaffected. There was no evidence of initial serostatus affecting the responses induced by the Salk IPV to the Sabin type 1 and 3. However, the GMTs of responses to Salk IPV assayed using Sabin type 2 virus were lower in initially seropositive infants (174, 95% CI: 112–271, n = 35) than in initially seronegative children (621, 95% CI: 430–898, n = 55).

When neutralization assays were performed using Salk viruses as assay targets there was little evidence of the initial serostatus affecting the responses to Salk types 1 and 3, but this was complicated by the generally poor responses in the sIPV groups. However, there was evidence of lower responses to the Salk IPV against the Salk type 2, with GMTs of 230 (95% CI: 136–391, n = 23) and 611 (95% CI: 464–806, n = 30) in initially seropositive and seronegative infants, respectively.

## 4 Discussion

This study was designed to perform an initial assessment of the safety and immunogenicity in infants of different dosages of a novel IPV candidate developed using Sabin viruses in a new manufacturing process designed to increase capacity at lower cost. Safety and tolerability of this sIPV were initially confirmed in fully vaccinated adults and then toddlers, before an independent DMC allowed progression to a dosage-finding study in infants. Three different dosages of the experimental sIPV formulation and a licensed Salk comparator IPV were then administered to infants in a three-dose schedule at 6,10,14 weeks of age. This confirmed the overall safety and acceptable tolerability of all three sIPV dosages, with no vaccine-related SAEs and a reactogenicity profile comparable to a licensed Salk vaccine.

In primed toddlers with a history of a complete three-dose primary polio vaccination series most subjects displayed immune responses (seroconversion) to a booster dose of *high*-dosage sIPV, and 100% seroprotection was achieved against all three serotypes using either Sabin or Salk viruses in the neutralizing assay. Post-booster GMTs for all three serotypes with either Sabin or Salk viruses were indistinguishable following vaccination with one dose of *high*-dosage sIPV or reference Salk IPV (Fig. 1).

However, in infants immunogenicity of sIPV was inferior to that of the Salk IPV with lower levels of immunity post-immunization with *low*- and *medium*-dosages of sIPV against the homologous Sabin serotype 1 and 2. This inferiority was more pronounced against the heterologous Salk serotypes. Although the *high*-dosage sIPV elicited similar responses to the Salk IPV, seroconversion rates with *low*- and *medium*-dosages of sIPV were poor for Salk serotypes 1 and 2. In contrast, the Salk IPV did not display any lower immunogenicity when using Sabin viruses in the assay. The immune responses against Sabin and Salk serotype 3 with all three sIPV dosages were comparable with the Salk IPV.

An important factor likely to influence the responses in our study was the young age of the participants. Vaccination was started at 6–8 weeks of age with a mean age of 6.5 weeks, when there were high levels of maternal antibodies, evidenced by the high baseline seropositivity rates, in particular for serotypes 1 and 2. Other recent studies in Panama found a similar profile of prevaccination seropositivity rates (47.6–52.2%, 51.9–62.6% and 20.6–24.2% for Salk virus types-1, -2 and -3, respectively) [22] and 60.7% for Salk type-2 only [23], due to maternal antibodies in 6 week-old infants. Interference by maternal antibodies with the responses to IPV has been established, [24], [25], [26], [27], [28], [29], [30], [31], and acknowledged by the WHO, the SAGE recommending that when a single dose of IPV is given in the mixed bOPV and IPV series it should be administered at 14 weeks of age [32]. A Puerto Rican study directly compared responses to Salk IPV administered in either the 6,10,14 weeks or 2,4,6 months schedules and found responses to types-1 and -2 were significantly lower with the earlier schedule [28]. A Cuban study reported sub-optimal immunogenicity with Salk IPV in the same schedule [32]. Our use of the 6,10,14 weeks schedule may partly explain the discrepancy between our results and those of other groups assessing similar sIPVs in less stringent primary immunization schedules. In China a 2,3,4 months schedule was used in their phase II studies [12], [13], while in the phase III study infants had a mean age of 10–11 weeks when receiving their first dose followed by two further doses one month apart, more closely approximating a 3,4,5 months schedule [14].

When we assessed GMT responses according to initial serostatus, the presence or absence of maternal antibodies revealed a clear suppression of the responses to types 1 and 2, but no apparent effect on type 3. This may indicate that candidate sIPV type-3 is sufficiently immunogenic to overcome interference with maternal antibodies, but it was notable that type-3 seropositivity due to maternal Abs was generally lower compared with types-1 and −2. This was most evident using Sabin viruses in the assay, and reflects the same observation made in the Puerto Rican study [28] for Salk vaccine and more recently by Tang et al [30] who also found that Sabin types 1 and 2 were most sensitive to this apparent interference, and the lower type-2 responses to a single dose of IPV at 16 weeks of age in seropositive infants [29]. The mothers of these infants would have been immunized with Sabin OPV, which was the standard of care in Panama until 2014 when the three infant primary doses were replaced by IPV. One may speculate therefore, that the maternal antibodies were specifically targeted against Sabin poliovirus antigens, including those presented in Sabin IPV and so cause more interference. Further investigation would be required to test this theory.

Responses to the reference Salk IPV were less sensitive to this apparent interference, and the Salk vaccine consistently induced higher immune responses against both Sabin and Salk strains. Therefore, there remains a discrepancy between the sIPV and the Salk IPV, even if lower responses were partially due to maternal antibody interference. Furthermore, the pattern of responses to the two vaccines was different with the Salk IPV eliciting high responses after only two doses, which were only matched by *high*-dosage sIPV after a third dose. Immunization against polio was based on OPV in the study area until 2014. It is therefore likely that all mothers of participating infants had been immunized with Sabin strains only. We can speculate on the possibility that OPV-induced maternal Abs selectively interfered more with sIPV vaccination and to a lesser extent with Salk IPV vaccination which results in a low immune response against sIPV when given at an early age with high maternal antibody levels.

Sabin strains of the same origin (JPRI) used in this sIPV candidate are currently used in a licensed combination DTaP-sIPV vaccine in Japan [33]. The Takeda sIPV has previously been tested in a dosage-ranging assessment in unprimed Japanese children at 3–67 months of age in whom they were administered as part of a DTaP-combination [16]. This older age range would not be expected to show any significant interference due to persisting circulation of relevant levels of maternal antibodies. Indeed, each of the same three dosages that we used as standalone vaccines, when administered as three DTaP-sIPV vaccinations, elicited dose-dependent increases in titers against all three homologous Sabin viruses that resulted in 100% seroprotection to all three serotypes. Responses to heterologous Salk strain viruses were lower quantitatively, but still achieved 93.0–100% seroprotection against the three serotypes across the *low*- to *high*-dosage formulations. Our use as stand-alone vaccine would not benefit from the inherent adjuvanticity of the DTaP component of the combination. Aluminium hydroxide was included as a stabilizer and adjuvant in the stand-alone formulation, but this did not improve the immune response in comparison with the unadjuvanted reference Salk IPV.

sIPV-induced antibodies have been shown to be cross-reactive against almost all poliovirus strains tested – Sabin, Salk, cVDPV and wild-type – the exception being an isolate excreted from an immunodeficient individual (iVDPV) [34]. In a collaborative study to establish standards for sIPV there were some small variations in antigen-specificity between Sabin and other polioviruses used by different manufacturers, including those used by Takeda [35]. However, these variations were too small to account for the low responses we observed.

Modification of the viruses during production would be expected to be detected in the preclinical assessments and lot release testing which have been standardized in agreement with other laboratories [35]. All vaccines used in the present study met all criteria in these lot release procedures, including animal (rat) immunogenicity testing in which the Sabin and Salk vaccines displayed similar immunological characteristics. Parents of infants who were low responders to the sIPV formulations were offered follow-up vaccinations with the licensed Salk IPV to ensure their child had full immunologic protection against polioviruses. Further laboratory investigations are underway to determine potential physical or chemical reasons for the low immune responses to the final clinical lots of Sabin preparation in human infants.

In summary, three dosages of experimental Sabin-based inactivated poliovirus vaccine were found to be well tolerated in vaccinees from 6 weeks of age through adulthood. However, immunogenicity in the youngest participants was negatively influenced by the presence of maternal antibodies and responses were inferior to a reference Salk IPV.

## Declaration of Competing Interest

The authors declare that they have no known competing financial interests or personal relationships that could have appeared to influence the work reported in this paper.
